# Integration of Point-of-Care Technology in the Decoding Process of Single Nucleotide Polymorphism for Healthcare Application †

**DOI:** 10.3390/mi16101159

**Published:** 2025-10-13

**Authors:** Thi Ngoc Diep Trinh, Hanh An Nguyen, Nguyen Pham Anh Thi, Thi Xuan Tuy Ho, Kieu The Loan Trinh, Nguyen Khoi Song Tran

**Affiliations:** 1 Biotechnology Institute, Tra Vinh University, Vinh Long 98000, Vietnam; 2 Department of Molecular Biology, Institute of Food and Biotechnology, Can Tho University, Can Tho 95000, Vietnam; 3 Institute for Global Health Innovations, Duy Tan University, Da Nang 58000, Vietnam; 4 Advanced Materials Technology Institute, Vietnam National University Ho Chi Minh City, Ho Chi Minh City 70000, Vietnam; 5 Vietnam National University, Ho Chi Minh City 70000, Vietnam; 6 NTT Hi-Tech Institute, Nguyen Tat Thanh University, District 09, Ho Chi Minh City 70000, Vietnam; 7 Nguyen Tat Thanh University Center for Hi-Tech Development, Saigon Hi-Tech Park, Ho Chi Minh City 70000, Vietnam

**Keywords:** loop-mediated isothermal amplification, polymerase chain reaction, point-of-care testing, recombinase polymerase amplification, single nucleotide polymorphism

## Abstract

Single nucleotide polymorphism (SNP) involves plenty of genetic disorders in organisms that can be passed down to the next generation or cause the stimulant signal that leads to early mortality in infants, especially within humankind. In medical field, real-time polymerase chain reaction (RT-PCR) is the most popular method for disease diagnosis. The investigation of genetic maps for the prediction of inherited illnesses needs the collaboration of sequencing technique and genome analysis. Although these methods are popular now, the cost for each test is quite high. Moreover, there is the requirement of extra machines and skillful technician or specialist level. Among these popular methods, the allele-specific polymerase chain reaction (AS-PCR), allele-specific loop isothermal mediated amplification (AS-LAMP), and allele-specific recombinase polymerase amplification (AS-RPA) are brought up for screening the nucleotide differences in the genetic sequence which will be noticed in this review as their availability, novelty, and potential for quick distinguishing of disease caused by SNP. Point-of-care testing (POCT) is a system built in a portable size but can perform the entire process of SNP recognition. Along with that, the POCT is intersected with the mentioned amplification methods and the genetic material preparation steps to become a united framework for higher efficiency and accuracy and lower cost. According to that, this review will focus on three common amplification techniques and their combination with POCT in the upstream and downstream process to genotype SNP related to human diseases.

## 1. Introduction

The establishment of various phenotypes of one species in the world is well explained, that is the result of the interaction between the genome and the living condition of the individual and the exposing of the inner genome sequence as the differences in the arrangement of the nucleotide. Single nucleotide polymorphism (SNP) genotyping is a critical field in biotechnology as the deletion, reversion, or replication of a single nucleotide is not only able to differentiate appearance and behavior for humans, animals, plants, or microorganisms but also can cause symptoms or diseases from mild to severe levels [1]. The necessity of SNP recognition is impossible to deny, which is why numerous methods are developed for rapid detection with high accuracy based on the amplification mechanism of advanced technology [2,3]. Recently, the outstanding development of sequencing technology has given researchers more aspects in human genomics due to the illustration of whole genome sequences or partition genome sequences such as partitional codon sequences, scaffolds, or contigs based on the desired and the capability of the sequencing technique [4,5]. There are two common types of sequencing, which are Sanger sequencing and next-generation sequencing (Illumia sequencing). They are classified based on the working mechanism as Sanger has high accuracy for short sequencing and less turnaround time referring to small target genome. Meanwhile, next-generation sequencing can perform simultaneously the varying of sequencing at the same time, longer waiting time, and suitable for huge genome [6,7]. Despite the ultimate function and capability of sequencing technology, it is still hard for wide application in low-resource areas because of the pricey reagent and machine for sequencing performance. Although X-crystallography has similar drawbacks with the sequencing method in the cost spending, the 3D structure of DNA and protein are apparently exhibited; therefore, the differences of the insight structure because of the SNP mismatch can be observed [8]. Due to the high sensitivity and stable reagents, amplification methods which recognize the mismatched bases of nucleotide variance are widely applied. Commonly, allele-specific (AS) amplification efficiently discriminates SNP by allele-specific (AS) PCR in a standard PCR reaction. The method proved to have high potential to be employed for clinical laboratories. The working principle is based on the amplification of nucleic acid triggered by effective primer when it matches its target. Amplification methods applied for SNP discrimination are rapid, sensitive, accurate, and cost effective and require common instruments found in standard laboratories. Currently, to optimize the application of amplification methods for SNP screening in more scenarios such as low-resource areas, the integration of point-of-care testing technology has been intensively studied. A reliable detection of SNP is expected to aid for the early medical intervention. Many approaches have been developed to achieve a reliable detection without the need for bulky equipment and trained personnel in a short time analysis. The application of point-of-care technology (POCT) meets the requirement for SNP detection at the patient site in short time. The incorporation of nucleic acid amplification technique with POCT platforms provides significant prospects for genetic screening due to its high selectivity and sensitivity. It also further improves the performance of amplification assay, making SNP genotyping more efficient, user friendly, and accessible. Besides simplicity, low cost, and high speed, the approach can facilitate multiple SNPs screening through expanding the multiplex ability of the test.

In this study, there are three core amplification methods named polymerase chain reaction (PCR), loop-mediated isothermal amplification (LAMP), and recombinase polymerase amplification (RPA) that will be carefully deliberated for their unique characteristics following with the suitable and bright developments for SNP classification. The main difference between PCR, LAMP, and RPA is the temperature operation of each method. PCR requires three different temperatures in a basic running for denaturation, annealing, and extension (elongation) stage while LAMP and RPA only need an unchangeable temperature in a range 60–65 °C and 25–42 °C, respectively [9]. With PCR, the concept of allele specific (AS) is a chart-topping technique for clarification of nucleotide mismatch which was first established for PCR in the 19th century, after which it was applied for LAMP and RPA as these two methods were found in the early 2000s [10]. Up to now, AS-PCR, AS-LAMP, and AS-RPA have been the three main basic platforms for various branches of improvements for SNP analysis that will also be rigorously selected and mentioned in each specific section of this review. On the other hand, POCT is an attractive topic in the contemporary world because of its characteristics such as high productivity, being lightweight for easy transport, and being economical to support any remote areas. It is widely known that POCT took the critical part in the prevention of coronavirus as well as COVID-19 by quick and accurate detection of the positive patient from raw swab samples of patients [11,12,13]. Hence, this paper hopes to illustrate an overview of POCT and selected amplification methods for SNP distinction in human diseases as much as possible, providing basic knowledge and updated information for readers and related scientists.

## 2. Common Methods for Genetic Material Preparation

Recently, there have been various methods for genomic isolation based on the type of genomic derivation and the specific purpose of the scientists. However, in the point-of-care testing (POCT) field, some significant ways are introduced to effectively extract the DNA from different types of samples. Herein, the extraction process is combined into an all-in-one companion device [14] or complete machine [15]. Microfluidics, one of the subfields in POCT, has the potential for DNA extraction. However, microfluidic devices have an impactful drawback in the sense that they mostly require an essential force to perform. Therefore, in 2015, Han et al. innovated the self-powered switch-controlled nucleic acid extraction system (SSNES) using an automatic regulative generating force for pulling the prepared samples and reagents through a microchannel with a vacuum actuator and a Venus-shape symbol object acting as a switch to manage power [16]. This system achieved low-cost manufacturing because of the majority of assembly of expendable syringes and accessible plastic. Nucleic acid from urine is gathered in the first step by APTES (3-Aminopropyltriethoxysilane)-treated binding channel when the obtained DNA/DMA (DNA/dimethyl adipimidate) complexes after the urine are lysed in the chemical mixture flow through. The unnecessary molecules in the microchannel are removed with a PBS buffer injected with the second reservoir in the next step, and then the amine bond between DNA and APTES with DMA is split with an elution buffer. The eluted DNA is amplified by using conventional PCR testing with the desired bladder cancer biomarkers and is illustrated with clear bands after gel electrophoresis analysis.

Petralia et al. introduced a new way in the later year for extracting DNA with a microfluidic device utilizing constructed silicon micropillars as DNA chromatography [17]. They conscientiously modified the micropillars in the channel to glean the highest SVR (surface-to-ratio), meaning that most DNA is bound. They found that the applicable solution for elution is purified water which can easily be found in any laboratory. The extraction productivity of genetic material of the HBV samples by using the device from Salvatore and his colleagues’ hits 40% providing a 16% higher value than a commercial kit in their measurement under the same condition. Additionally, the whole process of nucleic acid extraction from the cells and blood samples can happen in the tube with magnetic bead and the segment-injected buffer which correspond to each function such as lysis, washing, and elution [18]. Additionally, the microfluidic concept is implemented in the liquid phase and solid phase of DNA extraction. Govindarajan et al. launched a fabricable paper-phase device integrating microfluidics for the capability to prepare the nucleic acid from 60 to 90 min. Their approach brought a new approach to biological fields since they successfully collected DNA from slimy samples using not only the foldable paper-based device to allow the interactions between samples and reagents but also the column chromatography for DNA made of a stack of paper [19]. In addition, a group researcher freely skipped the preparation step from oropharyngeal and nasal swab samples with modified nanomaterial to detect the SARS-CoV-2 so the whole protocol does not take too much time, and one is able to observe the result with naked eye [20]. Integration of these extraction strategies with microfluidic systems is particularly valuable for advancing point-of-care (POC) diagnostics. Silicon micropillars, when embedded in microchannels, provide a high surface-to-volume ratio within a controlled microfluidic environment, allowing efficient DNA capture and elution with minimal reagent use. Their compatibility with standard microfabrication makes them promising for compact and scalable lab-on-chip platforms. Magnetic beads, on the other hand, are easily manipulated by external magnetic fields within microfluidic chambers, enabling automated nucleic acid extraction steps (lysis, washing, and elution) without the need for bulky centrifugation. This magnetic bead-based integration is especially advantageous for POC devices, as it simplifies workflow and reduces operator dependency. Paper-based microfluidic devices further enhance accessibility by leveraging capillary action for fluid transport, eliminating the need for pumps or external power. Their foldable and low-cost format makes them highly suitable for resource-limited settings while maintaining compatibility with downstream amplification or detection modules. Collectively, these approaches illustrate how microfluidics not only miniaturizes and automates nucleic acid preparation but also makes extraction more portable, rapid, and affordable, thereby directly supporting the development of next-generation POC diagnostic systems.

## 3. Allele-Specific Polymerase Chain Reaction Amplification (AS-PCR)

It is clearly known that SNP contributes vastly to the biological diversity of many kinds of living organisms from micro to macro. To obviously distinguish the SNP, the amplification of the specific target sequence is more feasible and economical than the sequencing process. On the other hand, the amplification of a desired genomic sequence is appropriate for POCT. There are some prevalent methods for using amplification to SNP genotyping on the portable devices including PCR, LAMP, and RPA. Due to the booming aura of the PCR in late 1900s, allele-specific PCR (AS-PCR) was a noticeable concept and much perceptible research by Okayama and his team applied this technique consisting of discovering the deficiency mechanism of single-base substitution, insertion, and deletion mutation in the ⍺1-antitrypsin [21]. The confirmation for the duplex pattern of the primers in a region of the *HIV-1 gag* gene was studied by Kwok and his colleagues [22]. In 1990, the incorporation of Ehlen modification into AS-PCR to examine the gene sequence of three polymorphisms in a single sperm at a locus was accomplished in Li’s lab [23]. In the 21st century, there have been plenty of dissimilar brilliant strategies for evolving the allele-specific PCR concept, becoming less time and chemically consuming. In favour of discovering multiple SNP and reducing genetic markers in AS-PCR, Bundock et al. constructed a simple approach to clarify the polymorphism of *H. vulgare* and *Hordeum spontaneum*, which is a three-primer nested system [24].

According to the AS-PCR concepts, they designed three primers including two forward and one reverse primer which means the inner forward contains the SNP genotype at the 3′ location. Their development contributed a huge understanding in the two groups of the Barley along with the confirmation of a large amount of nucleotide differentiation, specifically 36 SNP sites in 29 genes. While Alzheimer’s symptoms are ordinarily recognized by those possessing APOE4, one of three popular isoforms of APOE genes, it is widely believed that the combination of many gene factors with APOE gene isoform is the crucial point for the awareness of Alzheimer’s mechanism. Thus, the genotyping of five popular genes on the Alzgene database including *BIN1*, *CLU*, *ABCA7*, *CR1*, and *PICALM* became the main research topic with using two-times PCR in Darawi’s team in 2013 [25]. Their approach can show the SNP following the differences in the size of band on the gel and is able to do in any genetic laboratories as it only needs the essential equipment like a PCR machine and a gel documentation system, excluding the staining nucleic acid with fluorescent or hybridisation probes. In 2020, He et al. proposed the categorisation method of *ALDH2* gene (encoding aldehyde dehydrogenase 2) polymorphism implementing AS-PCR theory with methylene blue and the signals of fluorescence are measured with low-cost, highly sensitive, and small electrochemical sensor [26]. AS-PCR has turned into a backbone technique for the detection of SNP genotype six years since the PCR method was first published, which still has a lot of promising opportunities for development in future research.

## 4. Allele-Specific Loop-Mediated Isothermal Amplification (AS-LAMP)

Loop-mediated isothermal amplification (LAMP) is an amplificationmethod for genetic materials such as RNA and DNA within 15 to 50 min at an unchangeable temperature (about 60 to 65 °C), achieving the high efficiency of output result. Moreover, LAMP technique is often linked with colorimetric detection method for a probable-interpreting result in normal conditions without any equipment. Then, LAMP converges the necessary characteristics to work harmoniously with portable inexpensive chip devices for rapid spotting the target DNA and SNP genotyping in the samples. Based on the definition of AS-LAMP, Yongkiettrakul and her colleagues provided a proper manner for clustering the mutation points in *dhfr-ts* (dihydrofolate reductase of the bifunctional dihydrofolate reductase-thymidylate synthase) gene corresponding to medicine tolerance phenotypes in malaria parasites in 2017 [27]. They focused on the drafting of inner primers in LAMP, namely FIB (forward inner primer) and BIP (backward inner primer), for the recognition of SNP referring to the nucleotide at 5′ location. This manner propitiously distinguished the N511 mutation, but it cannot clarify well the wildtype (S108) and quadruple mutant (N108) of the target gene.

**Figure 1:**
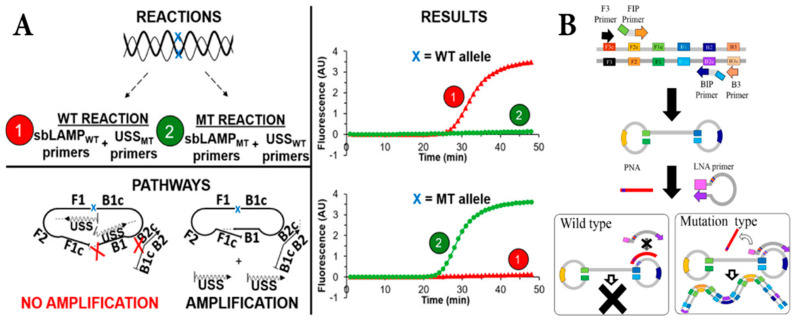
The schematic view of USS primer (**A**) and PNA–LNA cofactor. Copyright ACS Publications (2018) [28]; (**B**) in AS-LAMP. Copyright PLOS (2016) [29].

In 2018, Cardenas et al., illustrated the new approach of AS-LAMP about the primer strategies to strengthen the SNP detection including SNP-based loop mediated isothermal amplification (sbLAMP) primers and the unmodified self-stabilizing (USS) competitive primer (Figure 1A). They designed 6 sbLAMP primers for aiming 8 different sites and a USS primer set composed of a forward blocking competitive primer (FB) and a backward blocking competitive primer (BB) [28]. They established the SNP site at 5′ end of inner primers (sbFIP and sbFIP) on the F1c and B1c sections in the sbLAMP set, and USS primers take responsibility for enhancing the sensitivity and specificity and limiting the cross-binding of sbLAMP primers complex. Their study revealed the artemisinin-resistant SNPs in *C580Y* and *Y493H* genes with a low limit of detection, about 5 × 10^1^ copies, during 30 and 35 min, respectively. An exciting exploration of Itonaga and his groups for distinguishing the SNP mutation of KRAS gene by the association of LAMP with LNA (locked nucleic acid) and PNA (peptide nucleic acid) is widely published [29]. While the survey genes are amplified by the ordinary primers in LAMP technique, PNA and LNA are designed as the switch in the whole process of amplification (Figure 1B). Notwithstanding the conjugation of LNA in annealing and extension as the mutated DNA template, PNA will stop the multiplication of DNA forming a second layer at the dumbbell structure to prevent the activity of LNA if the DNA template is wild type. The given AS-LAMP assays have some points for the POC applications such as high sensitivity and specificity, short-time consuming, and constant temperature operation.

## 5. Allele-Specific Recombinase Polymerase Amplification (AS-RPA)

**Figure 2:**
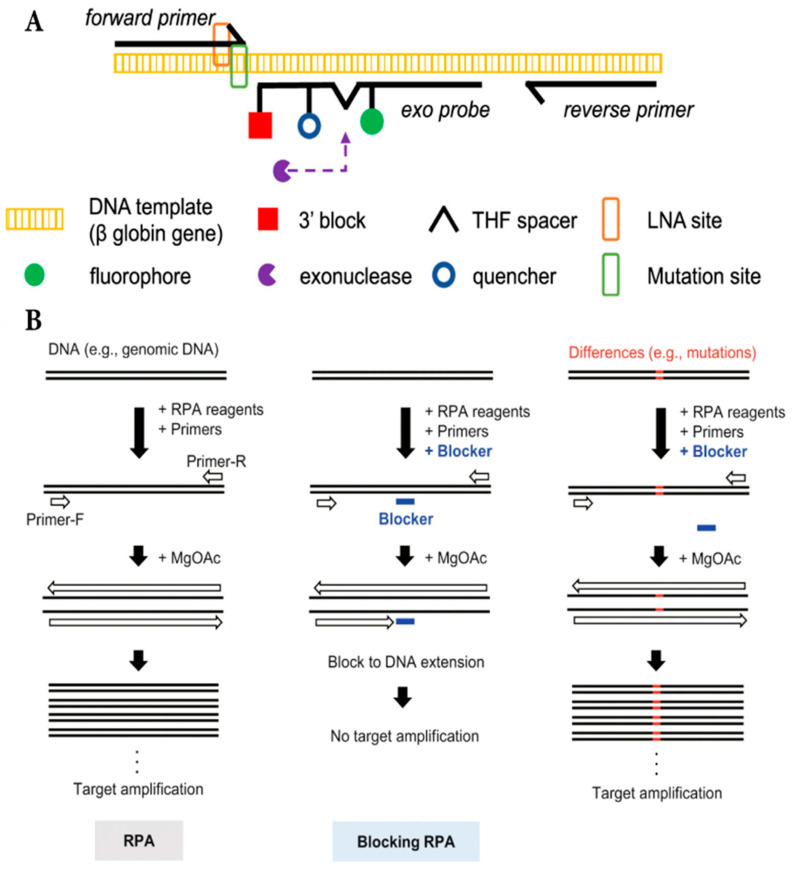
The general view of the AS-RPA assay. (**A**) The mechanism of LNA in RPA amplification. Copyright ACS Publications (2021) [30]. (**B**) The illustration of ORN as a blocker in the assay. Copyright Springer Nature (2021) [31].

Recombinase polymerase amplification, also known as RPA, is another isothermal amplification method that is found in the 21st century for analysis of RNA and DNA sequences. Although the temperature for RPA techniques is quite lower than LAMP, from 23 °C to 35 °C, RPA requires two principal reagents, which are recombinase enzyme and single-strand DNA binding protein (SSB), to perform correctly the amplification phenomenon in eukaryotic cells. Thus, the RPA has a huge impact on the unification of POCT, but it also needs skillful technicians and deep research as the concern of contamination. In 2021, Natoli et al., introduced AS-RPA with LNA to arrange the level of sickle cell disease due to the differences in nucleotide sequence from the patient’s blood sample and fast result delivery with fluorescent signal (Figure 2A). Their strategy applied LNA as a sorting machine to distinguish specifically the abnormal sequence in hemoglobin which clearly illustrates the appearance of allele S in the template sequence, and it contributed to the robustness of this system [30]. The amplification will be measured by the fluorescent signal reader since the amount of DNA amplicons is directly proportional to the intensity of fluorescence.

In the same year, Fujita et al. illustrated a new effective scheme of nucleotide discrimination with RPA and oligoribonucleotides (ORN) working as an inhibitor at the specific template sequence to detect nucleotide friction (Figure 2B) [31]. ORN is actually a small segment of RNA about 20 ribonucleotides long and it successfully inhibits the amplification of the KRAS gene as it attaches to the target gene for blocking the operation of RPA, which differs from Itonaga’s study. In this study, the researchers also compare ORN with other methods which are able to limit the RPA on purpose such as heat-sensitive protein, clustered regularly interspaced short palindromic repeats (CRISPR), oligodeoxyribonucleotide (ODN), and DNA-binding proteins. Although the ORN approach has some drawbacks including the easier biodegradability than ODN, the lower precision than CRISPR and DNA-binding protein technique, the ORN is easier to design than ODN and more cost-effective than CRISPR, and more pragmatic than DNA-binding protein. There is little research on the AS-RPA for SNP genotyping because this amplification performance was innovated in the early years of the 21st century but it has enormous potential to apply to POCT as its characteristics are quite the same as those of LAMP and it is a new shortcut to inherit the quintessence from the development of PCR and LAMP.

## 6. Point-of-Care Technology Applications for Allele-Specific Amplification

### 6.1. The Colorimetric Detection Methods Integrating to POCT System

In PCR assay, the most popular materials to detect the amplification product are nanoparticles, gold nanoparticle and silver nanoparticles particularly, according to their unique interaction with DNA in electrochemical force and feasible size to approach the amplicons [32,33]. With the appearance of the product in the successful amplification of PCR, the silver nanoparticles (AgNP) and gold nanoparticles (AuNP) will not be aggerated in the high concentration of sodium chloride due to the prevention of reducing repulsive interaction between the nanoparticle caused by sodium chloride of DNA. Recently, the surface of these nanoparticles has been modified with various biomolecules for improving the detection diversity. AgNP and AuNP are activated on the surface to be coupled with aptamer, short single strand of nucleic acid sequence, meditated with thiol group to detect heavy metal ions [34,35] and biomolecule [36,37]. The biomolecule capturing ability of hybrid nanoparticle probe is applied to some amplification methods and AuNP is the most commonly popular nanoparticle for the investigation in this aspect. The modified AuNPs are able to join in the amplification process and possess the characteristic to enhance the specificity of the PCR method for RNA and DNA target from simple [38,39] to advance [40].

Additionally, the AuNP and AgNP can also be used for the other amplifications such as LAMP and RPA for the colorimetric detection recognized with naked eye. The concept of using LAMP to detect some pathogenic bacteria and illustrating the amplification result with AuNP is commonly used [41]. To increase the specificity and sufficiency of this concept and avoid the contamination, some modifications are performed such as modified probes with glutathione [42], nanogold probe with uracile-DNA-glycosylase [43], and mimicking the protein surface with capping gold nanoparticles [44]. On the other hand, the amplicons can also be detected in low buffer due to the change in pH condition to lower pH for the complete amplification and make the shift of color in pH indicator. Based on that concept, the PCR product makes pH change to below 8.5 so the phenolphthalein [45] and phenol red [46] shows the color change. The successful LAMP process changes the pH of the buffer from 8.8 to around 6.5, which makes the color transition between the positive samples and negative samples of phenol red, neutral red, cresol red, and m-cresol purple [46,47]. The low pH buffer was also carefully investigated with RPA assay for the pH variations according to the PPi concentration shift and HCl titration [48] beside the development of colorimetric detection in RPA with the enzyme-linked oligonucleotide assay and surface-enhanced Raman scattering [49].

### 6.2. Typical Point-of-Care Systems

The provided amplification methods derived from the allele-specific theory to recognize the SNP in nucleic acid generated the variance in the allele-specific amplification methods. These methods have enormous potential for the integration with POCT which is able to reach any location and ethnicity in the world. Furthermore, these systems are usually associated with rapid outcome, and the result is obviously seen with bare eyes or common conditions, which are broadly known colorimetric detection approach. Table 1 shows the summary of the relative advantages and disadvantages of these amplification methods when integrated into POCT platforms.

**Figure 3:**
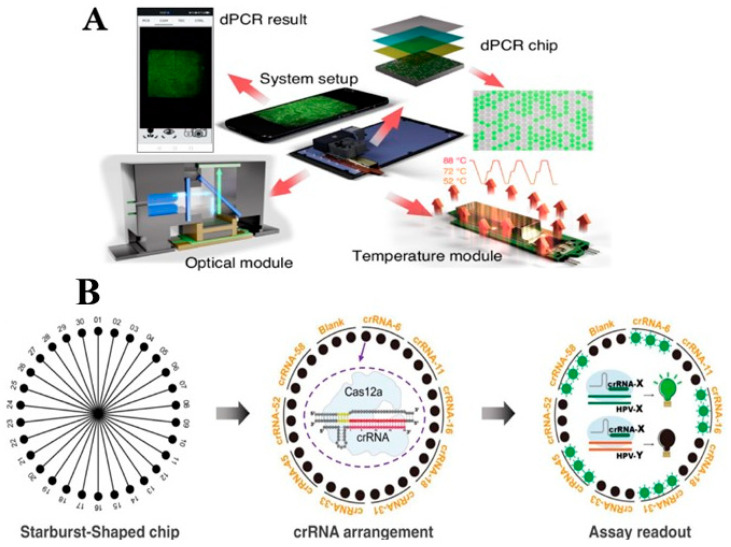
The overview of the POCT device. (**A**) The integrated portable system of smartphones and the fabricated thermal device for high throughput PCR. Copyright Springer Nature (2024) [53]. (**B**) The structure of MiCar device and its performance with AS-RPA. Copyright Springer Nature (2022) [58].

Although these platforms are made from replaceable and reasonable price materials, short-time consuming, and able to integrate with microarray chips [50] and capillary electrophoresis microdevice [51], they still have required some supplemental machines and special transportation. The compact DASH machine, which is based on PCR method and can detect the SARS-CoV-2 and its variants with high precision within 15 min, was commercialized and approved by FDA and EUA [52]. In 2024, Zhang and his colleagues published exciting research relating to the built-in POCT with a smartphone for PCR called SPEED; that means the system is controlled by the smartphone and the result can be read and analyzed by the smartphone owner (Figure 3A). They designed the PCR chip to perform high-throughput multiplex PCR at one time and a portable heating system with an electric circuit named thermoelectric element (TEC), which can simply work as a real PCR machine [53]. The surface of the polydimethylsiloxane (PDMS)-based PCR chip is 9 × 9 mm^2^, having 4096 micro-wells to perform the PCR and it is divided into six sections for image capturing which can be obviously observed on an android smartphone via Bluetooth or window monitor computer. This device is profitably utilized for COVID-19, pancreatic cancer target samples, and chromosomal disorders which emphasize its broad applicability for molecular diagnostics. LAMP assay is frequently used in POCT system due to its amplification condition. It can be used on the paper-based POCT platform [54] or slidable paper-integrated plastic microdevice [55]. Zhang et al. figured out the microcapillary system, known as icLAMP (integrated capillary LAMP), for classification of *CYP2C19* in raw blood samples. This system is capable of directly operating with untreated samples because of capturing DNA of FTA (Flinders Technology Associates) membrane and fast exposing under UV light as the luminescent feature of the fluorescent in the microcapillary binding to the DNA amplicons [56]. This approach can discriminate between heterozygous and homozygous types with a cheap price (0.2 $) and the detection is conceded to be observed with a portable UV lamp within 150 min. The extraction of DNA with FTA technology and the enlightenment of LAMP amplicon products with fluorescence under UV light were also placed into modified pipette tips by Lu and his colleagues in 2016. While the microcapillary system of Zhang can only use one sample at one time, the innovative pipette tip of Lu’s team can gracefully accommodate multichannel micropipette to build a high throughput system having high efficiency of amplification and SNP recognition [57].

**Table 1:** The summary of the relative advantages and disadvantages of these amplification methods when integrated into POCT platforms.

Amplification Methods	Advantages	Disadvantages	References
**PCR**	▪ Reproducibility ▪ Quantitative diagnostic ▪ High specificity ▪ Multiplex detection ▪ Reducing assay time (30–90 min) ▪ Suitable for integrating with AI	▪ High cost ▪ Limitations for colorimetric readout ▪ Requirement for the use of thermal cycler	[52,53]
**LAMP** **(Isothermal)** **(60–72 °C)**	▪ High sensitivity ▪ No requirement for the use of thermal cycler ▪ Reasonable price ▪ Low resource area setting application ▪ Easy colorimetric readout ▪ Reducing assay time (30–60 min) ▪ Suitable for integrating with AI	▪ No auto analysis support ▪ Complicated primer design for SNP detection ▪ Unable to perform multiplex amplification	[54,56,57]
**RPA** **(Isothermal)** **(37–42 °C)**	▪ No requirement for the use of thermal cycler (low temperature) ▪ Reducing readout time ▪ Low resource area setting application ▪ Suitable for integrating with AI ▪ Reducing assay time (~40 min)	▪ High cost ▪ Limited reagent kits ▪ Requirement for sample pre-treatment or preparation	[58,59,60]

PCR: polymerase chain reaction; LAMP: loop-mediated isothermal amplification; RPA: recombinase polymerase amplification; AI: artificial intelligence.

On the other hand, RPA is also an isothermal amplification method that Xu and his team combined with CRISPR-Cas12a and microfluidic technique to perform a new approach named MiCaR (Microfluidic device with CRISPR-Cas12a and multiplex RPA) for multifarious detection of HPV subtypes (Figure 3B) [58]. They invented a round-shaped microfluidic device with one big input chamber in the middle and 30 wells on the edge of the device which is already loaded with the customized CRISPR-Cas12a for each target type of HPV. Beside the equal separation of injected samples to the wells through microchannels, this system used a fluorophore quencher-labelled oligonucleotide as a reporter which reveals a bright fluorescent signal if the crRNA (CRISPR-RNA) matches the HPV DNA and vice versa. The analysis of this system takes only 40 min from the amplification step to the fluorescence readout step, and the specificity and sensitivity are 98.1% and 97.8%, respectively. This POCT field can be further developed in future in various ways including the integration of POCT and artificial intelligence like deep learning or machine learning [59], to revolutionize the traditional method with the modified material [60]. Moreover, the SNP detection method will be more diverse along with the improvement of the POCT field and from the upgrade amplification method.

### 6.3. Point-of-Care Systems Integrated with Allele-Specific Amplification for SNP Detection

The amplification methods provided gained much attention and were combined with POCT system to screen for SNP. A direct isothermal amplification system for SNP genotyping was developed [61]. In this approach, LAMP was applied to directly detect SNP. The polymorpholisms of aldehyde dehydrogenase-2 (ALDH2) Glu504Lys and methylenetetrahydrofolate reductase (MTHFR) C677T were assessed. ALDH2 is responsible for oxidation process of aldehyde in the liver. The polymorpholisms of ALDH2 can cause the change in susceptibility of ethanol intake which can possibly lead to alcoholism and other alcoholic complications. Meanwhile, MTHFR plays the main role in folate metabolism. The variation in MTHFR genes affects the activity of MTHFR enzymes and causes some disorders including neurological disorders, pregnancy complications, and cancer. In this study, the screening of two genes for SNP was obtained rapidly and conventionally using direct isothermal amplification. Isothermal amplification ensures the application of nucleic acid amplification in low-resource areas. In addition, to solve the issues regarding complicated procedures for nucleic extraction, direct LAMP was employed. In this approach, samples from various sources including saliva, buccal swab, dried blood spot, and whole blood were pretreated with NaOH and then directly applied for amplification. A rapid qualitative SNP detection of multiple targets was obtained within 30 min, which indicated the potential for application in SNP genotyping. A study combined multiplex ligation probe amplification (MLPA) and lateral flow assay (LFA) to detect SNP of ALDH2 gene [62]. In this approach, cost-effective method integrating MLPA with AuNPs-based LFA was applied for visual detection of ALDH2 polymorphism. MLPA is the technique that replies on hybridization, ligation, and PCR which offers precise determination of genetic variation at single nucleotide levels. By selective recognition of designed probes and target wild-type and mutant sequences, ligation reactions occur and amplification subsequently happens. The results were finally determined by the red lines on the FLA strips. The system obtained rapid screening of SNP with blood samples with high sensitivity of 1 ng. A system integrating all analyses assays into one-tube POCT platform for multiplex detection of SNPs [63]. In this approach, rolling circle amplification (RCA) was combined with CRISPR/Cas12a to simultaneously detect four SNPs. RCA is an isothermal amplification method that facilitates the amplification by a pair of primers and Phi29 DNA polymerase. As a result, multiple *Mycobacterium tuberculosis* resistant-related SNPs could be successfully detected in a rapid, simple, and POCT manner using one-tube reaction with RCA and CRISPR/Cas12a technology. The limit of detection of this approach was 10^4^ aM with high sensitivity and specificity with no cross reactivity. A POCT platform was developed to detect hair loss-related SNP [64]. In this study, the AS-LAMP reaction is optimized with temperature and time for achieving effective amplification of the target rs6152 polymorphism of androgen receptor gene which was associated with androgenetic alopecia resulting in hair loss. Furthermore, the platform enabled naked-eye readout of the result by using Schiff’s reagent-based detection. The platform identified SNP within 40 min with the detection limit of 1 pg/μL of DNA contained in human serum. These studies show the promising potential of POCT integrating with amplification method for screening SNPs which contribute to early diagnosis of diseases.

## 7. Conclusions and Future Perspectives

AS-PCR, AS-LAMP, and AS-RPA are three discussed amplification technologies in this review for nucleotide mismatch identification in human diseases. These combine with some renovated methods to enhance the specificity and sensitivity of the target amplification including AS-nested-PCR, AS-double-PCR, AS-LNA-LAMP, and AS-ORN-RPA. Although PCR is a classic amplification method which requires 3 or at least 2 temperatures corresponding to stages of amplification cycles so most of the research will focus on increasing the concentration of template DNA to detect the SNP on that template easier by the nested-primers, LAMP and RPA only need 1 steady temperature for amplification. Hence, many support components have been deeply studied such as LNA, PNA, and ORN, which perform as the claw machine to pick out the aimed sequence according to the construction of those components. In general view, PCR is not as convenient as LAMP or RPA, but it is more stable due to LAMP and RPA which are easily contaminated. With the integration of POCT, AS-PCR somehow turns to a compact system that escalates the output efficiency with showing on smartphone, reduces the material cost such as the investment for a formal PCR machine, and raises the diverse raw sample accessibility. For AS-LAMP and AS-RPA, the amplification process can be done in the capillary tube or modified pipette tips or the designed microfluidic chip which have the inexpensive price and reveal the fast result with a portable UV lamp.

Allele-specific PCR, LAMP, and RPA each present distinct advantages and limitations. PCR remains the gold standard for genotyping due to its high specificity and sensitivity; however, its reliance on precise thermal cycling and laboratory-grade equipment limits portability and increases operational complexity. In contrast, AS-LAMP operates at a constant moderate temperature (60–65 °C) and produces results within 30–50 min, often detectable by simple colorimetric readouts. Its robustness against inhibitors and compatibility with low-cost heating devices make it more suitable for resource-limited settings. AS-RPA offers even greater operational simplicity, as amplification can occur at ambient temperatures (23–37 °C) with minimal instrumentation, and reaction times are typically under 30 min. Nevertheless, RPA reagents are costly, and the method is more prone to nonspecific amplification without careful primer and probe design, which may compromise reproducibility. From an equipment perspective, LAMP and RPA are inherently better suited for POCT compared to PCR, as they require only simple heating or, in the case of RPA, no heating at all. In terms of sensitivity, all three methods can achieve detection in the range of 10–100 copies, but PCR and LAMP generally demonstrate higher specificity, while RPA offers the fastest turnaround. Taken together, AS-LAMP and AS-RPA provide complementary pathways toward portable SNP detection, with LAMP being more robust for field applications and RPA offering rapid, instrument-free amplification, whereas PCR remains more appropriate for centralized or confirmatory testing.

Recent advances in sample separation have shown promise in integrating this step with isothermal amplification, thereby moving toward sample-to-answer systems. While most of current POCT platforms provide qualitative or semi-quantitative outputs based on visual color change, the incorporation of fluorescence probes, smartphone-based image analysis, or electrochemical sensors can enable quantitative analysis with improved reproducibility. Taken together, the suitability of these technologies for POCT depends not only on reagent design and amplification efficiency, but also on how effectively they integrate upstream sample preparation and downstream signal readout. Achieving a seamless workflow from raw sample to interpretable result remains the key challenge and opportunity for future development of allele-specific amplification in POCT applications. To make the long story short, the POCT leads the whole long process to a minimal protocol because of the wrapping of everything into a system from the DNA extraction with microfluidic on solid, liquid, and paper phase to the multiplication of the target DNA step and analyze the outcome with a simple fluorescent reader machine or even a smartphone. Moreover, there are still some enlightening opportunities to upgrade the AS-PCR, AS-LAMP, and AS-RPA such as the isothermal amplification for AS-PCR, a single band for a target sequence for AS-LAMP, and a short primer with nucleotide mismatch at 3′ location for AS-RPA.
